# Evidence of a Cell Surface Role for Hsp90 Complex Proteins Mediating Neuroblast Migration in the Subventricular Zone

**DOI:** 10.3389/fncel.2017.00138

**Published:** 2017-05-17

**Authors:** Leo M. Miyakoshi, Diego Marques-Coelho, Luiz E. R. De Souza, Flavia R. S. Lima, Vilma R. Martins, Silvio M. Zanata, Cecilia Hedin-Pereira

**Affiliations:** ^1^Biophysics Institute Carlos Chagas Filho, Federal University of Rio de JaneiroRio de Janeiro, Brazil; ^2^Laboratory of Cellular NeuroAnatomy, Institute for Biomedical Sciences, Federal University of Rio de JaneiroRio de Janeiro, Brazil; ^3^Department of Basic Pathology, Federal University of ParanáParaná, Brazil; ^4^Institute for Biomedical Sciences, Federal University of Rio de JaneiroRio de Janeiro, Brazil; ^5^International Research Center, A.C. Camargo Cancer CenterSão Paulo, Brazil; ^6^VPPLR-Fundação Oswaldo Cruz (Fiocruz)Rio de Janeiro, Brazil

**Keywords:** neuroblast migration, rostral migratory stream, HSP90, HSP70, Hop/STI1

## Abstract

In most mammalian brains, the subventricular zone (SVZ) is a germinative layer that maintains neurogenic activity throughout adulthood. Neuronal precursors arising from this region migrate through the rostral migratory stream (RMS) and reach the olfactory bulbs where they differentiate and integrate into the local circuitry. Recently, studies have shown that heat shock proteins have an important role in cancer cell migration and blocking Hsp90 function was shown to hinder cell migration in the developing cerebellum. In this work, we hypothesize that chaperone complexes may have an important function regulating migration of neuronal precursors from the subventricular zone. Proteins from the Hsp90 complex are present in the postnatal SVZ as well as in the RMS. Using an *in vitro* SVZ explant model, we have demonstrated the expression of Hsp90 and Hop/STI1 by migrating neuroblasts. Treatment with antibodies against Hsp90 and co-chaperone Hop/STI1, as well as Hsp90 and Hsp70 inhibitors hinder neuroblast chain migration. Time-lapse videomicroscopy analysis revealed that cell motility and average migratory speed was decreased after exposure to both antibodies and inhibitors. Antibodies recognizing Hsp90, Hsp70, and Hop/STI1 were found bound to the membranes of cells from primary SVZ cultures and biotinylation assays demonstrated that Hsp70 and Hop/STI1 could be found on the external leaflet of neuroblast membranes. The latter could also be detected in conditioned medium samples obtained from cultivated SVZ cells. Our results suggest that chaperones Hsp90, Hsp70, and co-chaperone Hop/STI1, components of the Hsp90 complex, regulate SVZ neuroblast migration in a concerted manner through an extracellular mechanism.

## Introduction

During development, mechanisms that regulate neuronal migration are essential for the correct organization of the neural tissue. Defects in neuronal migration underlie neurological and psychiatric disorders. From early postnatal life throughout adulthood, neurogenic niches of the brain are restricted to mostly two main regions: the hippocampal dentate gyrus and the subventricular zone (SVZ) (Fuentealba et al., 2012). The migration of neuroblasts from the SVZ to the olfactory bulbs is a long migratory route, giving rise mostly to postnatal GABAergic interneurons (Luskin, 1993). The SVZ acts as a reservoir for new cells throughout lifespan in mammals. Therefore, studies concerning mechanisms of neurogenesis and migration in this region can provide the foundations for future cellular therapies for brain lesion repair. The movement of SVZ neuroblasts is characterized by a neuronophilic migration mode where neuroblasts migrate over each other in what is called chain migration (Lois et al., 1996).

Several molecules have been described to regulate this migratory stream toward the olfactory bulb (for reviews see, Menezes et al., 2002; Sun et al., 2010), however the control of proliferation and migration of SVZ neuroblasts is a complex network of molecular interactions that is still not completely understood. Some of these are guidance cues, attractive molecules produced by the olfactory bulbs, such as Netrin (Murase and Horwitz, 2002), and repulsive molecules produced by the septum, such as Slit (Wu et al., 1999; Nguyen-Ba-Charvet et al., 2004). In addition, the cell-cell interactions are also important for neuroblast migration. PSA-NCAM was suggested to have a role in maintaining migration by regulating the adhesion between cells. Extracellular matrix (ECM) elements, such as laminin, tenascins, proteoglycans, and reelin have been shown to be important mediators of cell-cell, cell-substrate interactions for neuroblast migration (Tomasiewicz et al., 1993; Ono et al., 1994; Chazal et al., 2000; Hack et al., 2002; Saghatelyan et al., 2004).

Chaperones and co-chaperones are well-known to act in concert to assist protein folding, protein degradation and other essential roles, such as the maintenance of protein conformations under normal or stress conditions (for review see, Wegele et al., 2004; Pearl and Prodromou, 2006). Recently non-classical roles have been proposed for Hsp90 acting on the cell surface of neural and cancer cells (Eustace and Jay, 2004; Sidera et al., 2004, 2008). Other components of the Hsp90 complex, such as Hsp70, co-chaperone Hop/STI1 (Hsp70/Hsp90 Heat-shock organizing protein/Stress inducible phosphoprotein 1) and p23 have been identified extracellularly (Zanata et al., 2002; Eustace and Jay, 2004; Lima et al., 2007; Sims et al., 2011) raising the question of whether these proteins might be acting as a complex outside the cell. On the other hand, Hop/STI1 has also been described as a secreted protein and an extracellular ligand (Hajj et al., 2013) to the cellular prion protein, participating in several neural developmental processes (Zanata et al., 2002; Lopes et al., 2005; Arantes et al., 2009; Beraldo et al., 2010; Santos et al., 2011). Hop/STI1 is a tetratricopeptide domain-containing co-chaperone that directly interacts with the C-terminal of Hsp90 (Young et al., 1998). Based on previous evidence of the cell surface or extracellular role of Hsp90 in migration of neural and cancer cells (Sidera et al., 2004, 2008), as well as the role of Hop/STI1 in promoting glioblastoma cell migration (Fonseca et al., 2012), we sought to investigate if an extracellular complex composed of Hsp90 and Hop/STI1 could be involved in SVZ neuroblast migratory behavior.

## Materials and methods

Animals in these experiments were Swiss mice obtained from the animal facility of the Institute for Biomedical Sciences of the Federal University of Rio de Janeiro. All experiments were performed in conformity with National Council for Control of Animal Experimentation (CONCEA, Brazil) guidelines for animal care and in accordance with protocol approved by the Committee of Ethics on Animal Handling and Care at the Federal University of Rio de Janeiro (CEUA/033-15; ICB/CCS—UFRJ).

### SVZ explant cultures

Similar to Miyakoshi et al. (2012), P0-P7 mice were sacrificed and brains removed from the head. Parasagittal slices 350 μm thick were obtained with a tissue chopper (Mcllwain) and anterior SVZ was dissected out under scope inspection and were further cut into small pieces of approximately 100 μm diameter. These explants were placed on 4-well plates (NUNC) with Neurobasal (Gibco) medium supplemented with B27 (1x), Penicillin/Streptomycin (1x), and Glutamine (2 mM) for 30 min. Next, medium was carefully withdrawn and Matrigel (Collaborative Biomedical Products) diluted in medium (1:3) was pipetted to cover all explants in each well. This ensures a three-dimensional environment suitable for neuroblasts to migrate out of explants.

### Immunohistochemistry

Early postnatal animals (P0-P7) were sacrificed and their brains were fixated through overnight PF 4% immersion. Brains were sectioned parasagitally (50–70 μm) in a vibratome (Vibratome 3000). The sections used to demonstrate the expression of Hsp90 and Hsp70 proteins were submitted to antigen retrieval procedures consisting of immersion in 10 mM Citrate Buffer at 90°C during 10 min, followed by 20 min in cold PBS 0.1 M. Before incubation with primary antibodies sections were washed in 0.3% PBS Triton X-100 and kept for at least 1 h in a room-temperature blocking solution (NGS 5% diluted in 0.3% PBS Triton X-100). After this period of time, sections were incubated overnight with either anti HOP/STI1 (Bethyl, 1:500), anti Hsp90 (Millipore AB3466, 1:50) or anti Hsp70 (Santa Cruz, SC-33575, 1:50). In the next day, sections were washed with PBS and incubated for at least 2 h in room temperature with Goat anti Rabbit Alexa 546 secondary antibodies (Invitrogen, A11010, 1:400). Finally, sections were again washed with PBS, submitted to nuclear staining with DAPI for 5 min and mounted in glass microscope slides with N-Propyl Gallate (Merck).

### Immunocytochemistry

First, cultures were exposed to 15 min of formaldehyde vapor and then immersed for 5 min in PFA 4%. Then wells were washed with PBS 0.1 M and maintained in NGS/PBS-T (5% goat serum and 0.3%Triton X-100) blocking solution for 1 h at room temperature. In sequence, rabbit anti-Hsp90α polyclonal antibody (1 mg/mL CHEMICON, AB3466), rabbit anti-Hsp90α (1 mg/mL Milipore, 07-2174) or anti-Hop/STI1 polyclonal antibody (Bethyl, rabbit 33, purified IgG 2.2 mg/mL, Zanata et al., 2002) were diluted in the same blocking solution, respectively at 1:50 or 1:500 and incubated overnight at 4°C. Sometimes in addition to chaperone immunolabeling, double labeling immunocytochemistry was performed with a monoclonal antibody against PSA-NCAM (CHEMICON, mAB5324) at a 1:200 dilution in blocking serum. After that, goat anti-rabbit secondary antibodies were used conjugated with ALEXA 488 (Invitrogen, A11008) or ALEXA 546 (Invitrogen, A11010) fluorescent markers at a 1:400 dilution. When mouse primary antibodies were used, goat anti-mouse CY3 (Jackson Labs, 115166003) at a 1:800 dilution or a goat-anti-mouse ALEXA 488 (Invitrogen, A11001) at a 1:400 dilution were applied.

### Immunoblocking and heat shock protein inhibition procedures

After 24 h (1 day *in vitro*, DIV), cultures were visualized in inverted microscope (Zeiss Axiovert) and checked for viability. Only wells displaying typical chain migration were selected and divided into control and experimental groups. In both groups, medium was replaced, however in the experimental groups, polyclonal antibodies recognizing Hsp90 (1 mg/mL, dilution 150 μg/mL-Milipore) and HOP/STI1 (2.2 μg/μL, Bethyl, rabbit 33, dilution 1:200; 0.011 μg/μL), as well as Hsp90 inhibitor Geldanamycin (100, 10, and 1 nM-Tocris Bioscience, 1368) and VER155008 (100 nM, 10 nM and 1 nM-Tocris Bioscience, 3803) were added to the medium. As a control, SVZ explants were treated anti-mouse IgG produced in Rabbit (Sigma-Aldrich A9044).

### SVZ migration

#### Halo measurements

At 1 DIV, Images were taken from explants displaying chain migration. After that, while control group had their medium replaced, treatment with antibodies and inhibitors was administrated in the experimental group. At (2 DIV), approximately 24 h after the onset of treatment, another set of photos were taken from the same explants. Quantitative evaluation was performed by image analysis using Image J software, which allowed us to obtain the perimeter around the explants. A migratory index, which consisted of the ratio between perimeter values obtained in the second and first day *in vitro* (2/1 DIV) was used to quantify migration in groups submitted to treatment as well as controls.

#### Time-lapse video microscopy

To quantify the average speed of neuroblasts, at 1 DIV, cultures were transferred to an inverted light microscope (Nikon Eclipse T200), suitable for live microscopy, equipped with an incubator with controlled temperature and gas exposure (37°C, 5% CO_2_). Selected fields containing chains of migratory neuroblasts were imaged every minute in a period that varied between 3 and 5 h. After that, also to extract quantifiable data, all images were analyzed in ImageJ software, where we manually tracked migrating cells (exclusively with neuroblast phenotype) and subsequently calculated distance/time (μm/h) to find average speed.

### Antibody internalization assay

Dissociated cells from SVZ were incubated before fixation with several concentrations of Hsp90, Hsp70, and HOP/STI1 antibodies for 2 h. After that cells were washed in DMEM and fixed in cold acetone for 3 min. To evaluate whether primary antibodies were internalized, cells were permeabilized with 0.1% Triton X-100 in PBS and subsequently incubated with secondary antibodies. In all experiments, we made a control group just using secondary antibody alone.

### Cell membrane fluorescence quantification

Fluorescence analysis and quantification were performed in Image J software. In order to quantify fluorescence in the cytoplasm as well in the cell membranes, we used the Image J software to draw a line that cut the cells transversally. Fluorescence intensity along this line was provided by the software and these values were then further quantified.

### Dissociated SVZ cell culture

As in SVZ explant cultures, the brains were extracted from P0-P7 mice and sectioned in 350 μm parasagittal slices, which were further submitted to dissection in order to obtain SVZ explants. Next, those pieces were gathered in a falcon tube and a soft mechanical dissociation was made until all pieces disappeared. To control how many cells would be plated, a Neubauer chamber (Optik Labor) was used, after which cells were plated in 5 mL flasks (3–4 × 10^6^/flask) or 40 mm plates (1–2 × 10^6^/plate). A DMEM medium supplemented with B27, Penicillin/streptomycin, glutamine and fetal bovine serum was used to maximize cell survival in the first 24 h.

### Biotinylation of SVZ primary cultures and pull down

After 1 DIV, medium was changed to a non-FBS DMEM supplemented medium. At 3 DIV, culture medium was removed and plates were washed with cold PBS (0.1 M and pH 8). Then, cell culture was incubated with biotin 2 mM (PIERCE) at 4°C for 45 min. Subsequently, cells were washed with glycine 300 mM for 10 min, to block and remove free biotins. We repeated this process 3 times. Then, cells were collected by scraping, pelleted (3.000 × g at 4°C) and mechanically homogenized with a syringe 26.6G in cold lysis buffer (Tris-Base 50 mM, sodium deoxycholate 0.2% and NP-40 1%) for 30 min in ice, supplemented with protease inhibitors cocktail (Sigma P8340) (1:200) and phosphatase inhibitors Na_3_VO_4_ 200 mM, NaF 1 mM. In sequence, samples were centrifuged at 14.000 × g for 10 min to remove cell debris and the supernatant is incubated with Avidin Beads (Thermo Scientific) for 1 h. The centrifugation and incubation with Avidin Beads was performed at 4°C.

Next, samples were centrifuged at 2.000 × g for 2 min and supernatant was reserved to later be used as negative control. Then, samples were washed with cold PBS solution 0.1 M ph 8.0 and centrifuged at 1.000 × g for 2 min at 4°C, this washing procedure was repeated 4 times. Biotinylated proteins remained attached to avidin in pellet. So as to remove the bound biotinylated molecules, samples were diluted in sample buffer (SDS-PAGE) and heated for 5 min at 95°C. Lastly, samples were centrifuged at 10.000 × g for 5 min and the supernatant, now containing the biotinylated proteins, is collected to proceed to western-blot.

### Conditioned medium of SVZ cell cultures

To collect conditioned medium samples from dissociated SVZ cell cultures, we removed the medium, washed culture flasks 3 times and added pure DMEM. After 72 h, cell culture viability was analyzed and conditioned medium collected. To enhance concentration of proteins, conditioned medium was submitted to lyophilization (SpeedVac Concentrator) through vacuum and centrifugation (about 24 h or until 10% of initial volume remains). Next, conditioned medium was stored at −20°C or prepared in loading buffer and applied in SDS-PAGE 10%.

### Western blotting

After the biotinylation/pull down or conditioned medium collection procedures, proteins from each extract were subjected to a gel eletroforesis (SDS-PAGE 10%) and transferred to a nitrocellulose membrane. The membrane was blocked for 1 h at room temperature with non-fat dry milk (5%) in TBS (Tris 0.02 M, NaCl 0.8%, diluted in water) and after that incubated overnight at 4°C with primary antibodies diluted in TBS. In the next day, membrane was washed with TBS-T (Tween 0.05% diluted in TBS) and incubated with secondary antibodies conjugated with peroxidase diluted in TBS-T. After washing with TBS-T and TBS, membrane was exposed to revelation reagents (Revelation kit Pierce) and taken for revelation in Chemidoc MP System (Bio-Rad).

## Results

### SVZ migratory neuroblasts express Hsp90 and HOP/STI1

Previous studies have demonstrated that chaperones Hsp90, Hsp70, Hsc70, and Hsp60 are expressed in rodent neural tissue and are regulated during postnatal development until the adult in the cerebellum, cerebral hemispheres and brain stem. Hsp90, 70, and Hsc70 are highly expressed in the first two postnatal weeks and have their expression decreased in P21 and adult (D'souza and Brown, 1998). Hsp60, on the other hand increases its expression toward adulthood. Co-chaperone HOP/STI1, an important component of the Hsp90 heterocomplex is also highly expressed in the brain from embryonic to postnatal ages (Hajj et al., 2009). However, the expression of these proteins in the SVZ and its neuroblasts has not been described. In order to characterize the presence of these chaperones and co-chaperones in migratory neuroblasts, we analyzed the expression pattern of proteins of the Hsp90 complex in the postnatal SVZ and rostral migratory stream (RMS) as well as in postnatal SVZ explant cultures by immunohistochemistry and immunocytochemistry, respectively.

**Figure 1:**
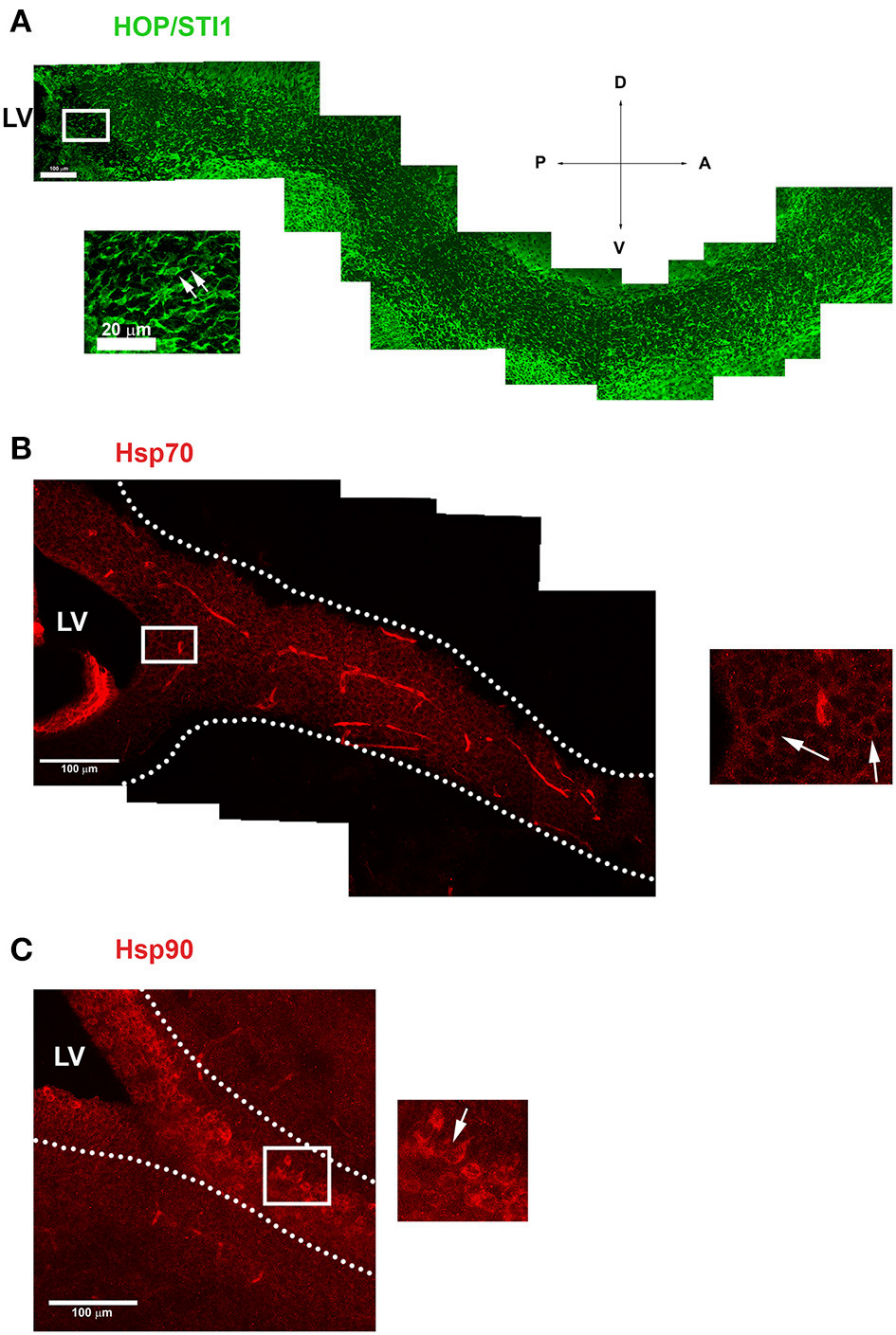
**Proteins from Hsp90 complex are expressed in the anterior subventricular zone and along the rostral migratory stream. (A)** Image demonstrates HOP/STI1 expression extending from the anterior portion of the lateral ventricle (LV) toward the olfactory bulb along the rostral migratory stream (RMS). Inset shows cells with typical migratory morphology in the vicinity of LV expressing HOP/STI1 (arrows). **(B,C)** Hsp70 and Hsp90 also exhibits a periventricular expression which continues along the RMS (delimited by dotted lines) In the insets, individual cells expressing these proteins can be visualized in the vicinity of the lateral ventricle **(B)** and along the RMS **(C)** (arrows). Scale bar: A(100 μm), inset of **(A)** 20 μm **(B,C)** 100 μm. LV-Lateral Ventricle. Wind Rose–D, Dorsal; V, Ventral; A, Anterior; P, Posterior.

**Figure 2:**
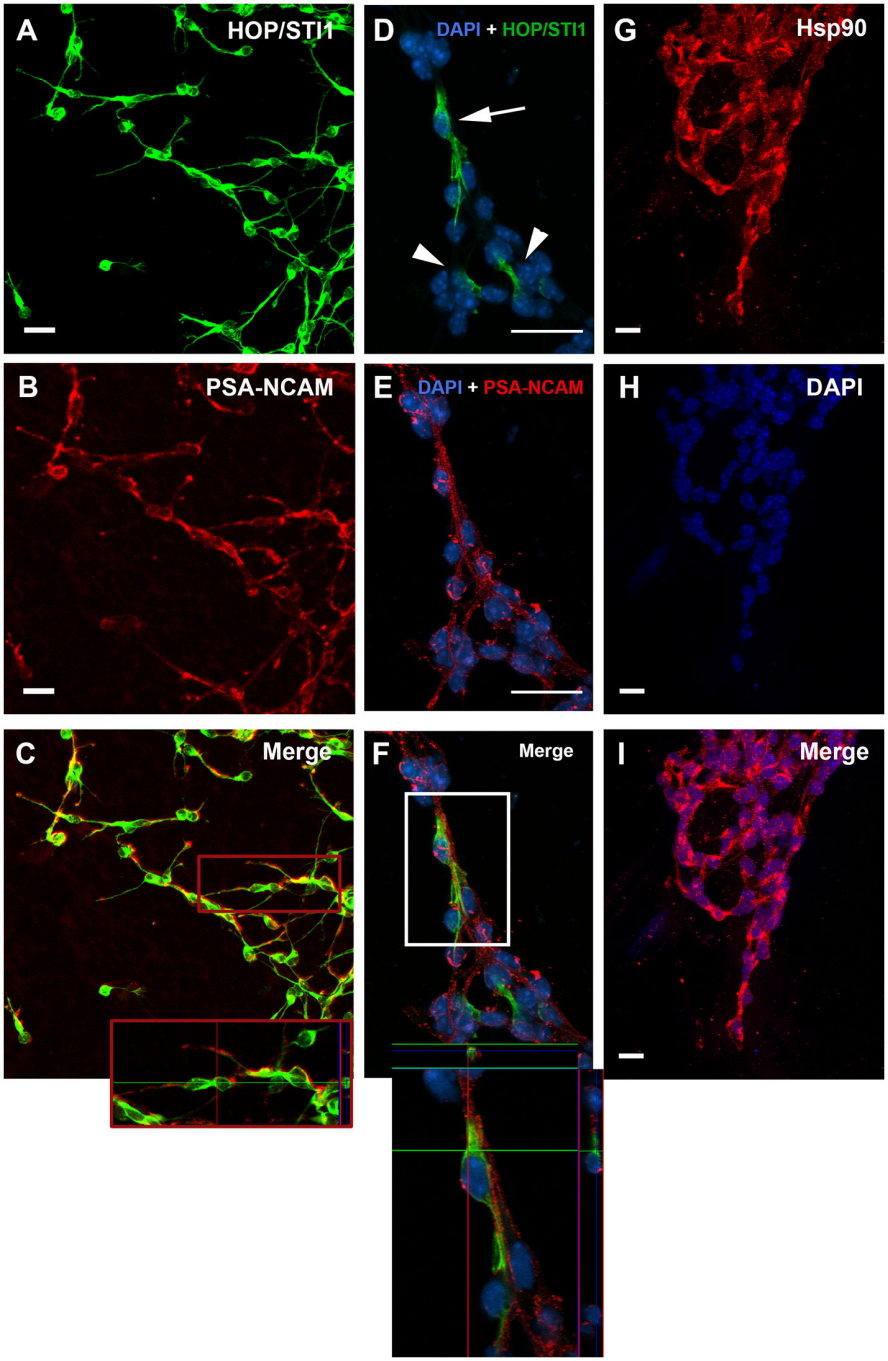
**HOP/STI1 and Hsp90 are both expressed by SVZ migratory neuroblasts. (A)** Migratory cells originating from SVZ explants immunolabeled against anti-HOP/STI1 antibody reveal an ubiquitous expression pattern. **(B)** PSA-NCAM expression is used to characterize neuroblasts from the SVZ. **(C)** Double labeling indicates that HOP/STI1 expressing cells are neuroblasts. Inset shows an orthogonal section depicting a chain of HOP/STI1^+^/PSA-NCAM^+^ cells. **(D)** HOP/STI1 is expressed predominantly in the cellular processes in non-permeabilized SVZ cultures (arrowheads). Only few cells within the migratory chains exhibit perinuclear expression (arrow). **(E)** Analysis of PSA-NCAM expression characterizes these cells as neuronal precursors that co-express HOP/STI1, as can be seen in more detail in an orthogonal section (**F**, Inset). In **(G)**, chains of migratory cells are also immunolabeled against anti Hsp90. In **(H)**, Dapi nuclear staining demonstrates these cells organized as chains, which is a hallmark of SVZ neuroblasts. Nuclear staining and Hsp90 expression are both depicted in the merge in **(I)**. Scale bars: **(A,B)** 20 μm, **(D,E)** 20 μm, and **(G–I)** 10 μm.

Parasagittal views allow the visualization of a continuous expression of HOP/STI1 (Figure 1A), Hsp70 (Figure 1B), and Hsp90 (Figure 1C) that extends from the periventricular area toward the rostral migratory stream. It is possible to identify individual cells in the vicinity of the lateral ventricle and in the RMS expressing these proteins (insets), some of these cells clearly exhibit leading processes, a hallmark of migratory neuroblasts (Figure 1A, inset, arrows). To explore the cellular level analysis even further, we employed an established explant model (Wichterle et al., 1997), which allows the formation of migratory neuroblast chains *in vitro* that exhibit a behavior similar to their *in vivo* counterparts. In this model, cells abundantly migrate from the explants in the first 24 h of culture. Migratory cells within chains were immunolabeled for anti-HOP/STI1 (Figure 2A) and anti-Hsp90 (Figures 2G–I) antibodies. The immunolabeling pattern was similar for HOP/STI1 and Hsp90, being present in cell membranes extending to the cell processes as well as in the cytoplasm around the nuclei. The migratory morphology and chain organization of labeled cells are typical of neuroblasts. However, when prior permeabilization of neuroblast membranes with triton-X-100 was omitted the expression pattern of HOP/STI1 changes in a drastic manner. In contrast to the ubiquitous distribution of the protein observed in Figure 2A, fewer cells within the migratory chains were HOP/STI1^+^. Although the perinuclear labeling is detected in some cells (Figure 2D, arrow), it is not as often as in permeabilized cultures. In most cases, HOP/STI1 labeling was restricted to neuroblast processes (Figure 2D, arrowheads). Despite the differences in the expression pattern, the cells maintain the migratory morphology and chain organization typical of neuroblasts (Figures 2A–F). Also, cells within chains co-expressed PSA-NCAM, indicating that they belong to the neuroblast lineage, which is a known marker of this population. Although triton x-100 permeabilization procedures clearly affected PSA-NCAM immunolabelling (Figure 2B), it is possible to observe that the HOP/STI1^+^ population also express PSA-NCAM (Figure 2C). A more accurate view of the PSA-NCAM expression pattern can be seen in the non-permeabilized cells (Figure 2E). In this case, the two markers can be detected in the same population, although there was no apparent co-localization in the membranes (Figure 2F, inset).

### Interfering with components of the Hsp90 complex, Hsp90, HOP/STI1, and Hsp70 disrupts neuroblast migration from SVZ explants

**Figure 3:**
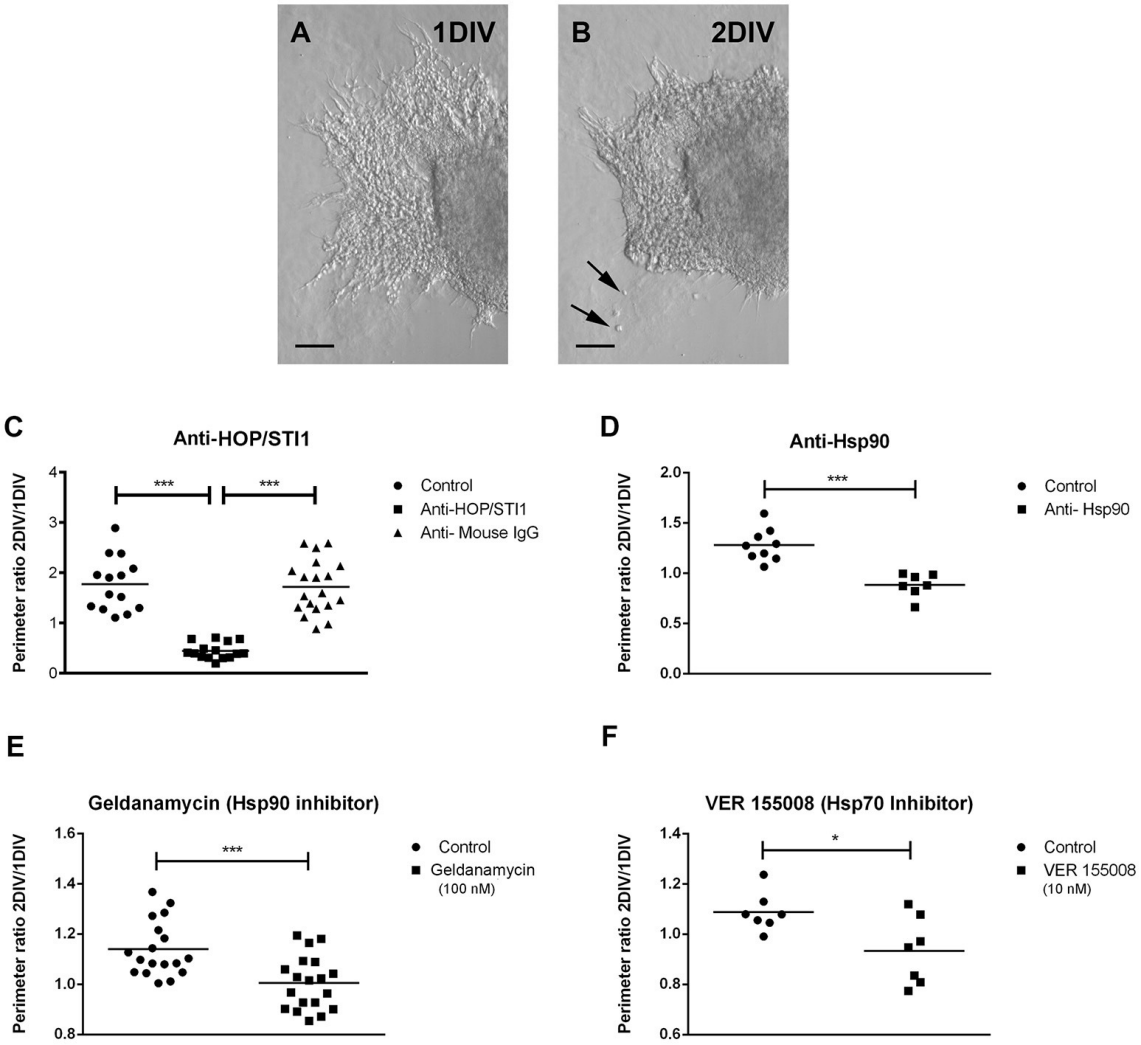
**Treatment with antibodies and inhibitors disrupt neuroblast chain migration from SVZ explants. (A)** Representative bright-field images depicting extensive neuroblast migration from SVZ explant culture at 1 DIV. **(B)** After antibody treatment (anti-HOP/STI1) at 2 DIV, a compact mass of cells is present in the vicinity of the explant and chain organization is practically lost. Some clusters of cells are detected in the sites previously occupied by the neuroblast chains (arrows). Antibody treatment reduces the perimeter of migratory halos around SVZ explants. In **(C)**, a significant reduction in the ratio of 2/1 DIV perimeter obtained from explants submitted to anti-HOP/STI1 treatment was detected when compared to both control and anti-Mouse IgG treated groups (Kruskal-Wallis test, Dunn *post-hoc* test, *p* < 0.0001). **(D)** A similar result was observed after treatment with anti-Hsp90 antibody (Unpaired *T*-Test, *p* < 0.001, *n* = 4). Migratory halos were also reduced after treatment with Hsp90 inhibitor Geldanamycin (**E**, unpaired *T*-test, *p* < 0.001) and VER155008, which inhibits Hsp70 (**F**, Mann-Whitney test, *p* < 0.05). ^*^p < 0.05, ^***^p < 0.001.

The expression pattern of these proteins indicates that they could play a functional role in this type of migration. To investigate this, cultures were treated at 1 DIV (24 h after plating) with specific antibodies to HOP/STI1 and Hsp90, as well as inhibitors to Hsp70 and Hsp90 (Figure 3). At 2 DIV, migratory halos, composed by chains of neuroblasts originating from the explants, were evaluated. Antibody treatment affects migratory chains which present severe morphological alterations. In most cases, the distal chains which shoot out at 1 DIV were no longer detectable; the migratory halos presented a compact organization, often forming clusters in the proximity of the explants (arrows in Figures 3A,B). We then quantified the effects of the different treatments on the migratory halos as described in Miyakoshi et al. (2012).

Shortly, the measurements of the perimeter of the migratory halos at 1 and 2 DIV were used to obtain a ratio (2/1 DIV) providing a migratory index for every explant. The measurement at 1 DIV was taken just before antibody and inhibitor treatment. Statistical analysis of these data revealed a significant decrease in the migratory index when cultures were exposed to anti-HOP/STI1 compared to cultures without treatment or treated with an antibody that recognizes irrelevant mouse IgG (Figure 3C, Kruskal Wallis test, Dunn's multiple comparison test was used as *post-hoc* test *p* < 0.0001). Since there was no statistical difference between the Control group and the one treated with anti-Mouse IgG, further experiments did not employ IgGs as a control. Quantitative analysis demonstrates a similar retraction in the migratory halos following exposure to anti-Hsp90 (Figure 2D, Unpaired *T*-Test, *p* < 0.001, *n* = 4). To test whether the effects of the antibodies were specific, we also analyzed the response of migratory cells to chaperone inhibitors. Corroborating the hypothesis of functional impairment mediated by antibody binding, the treatment with inhibitors to Hsp90 (Geldanamycin) and to Hsp70 (VER155008) induced similar alterations in the migration of neuroblasts, including the more compact organization of chains around the explants. Statistical analysis of the migratory index revealed significant reduction in migration from 1 to 2 DIV (Figures 2E,F. Geldanamycin treatment 100 nM, One-way ANOVA *p* < 0.01, *n* = 9: VER 155008 treatment 10 nM, One way ANOVA *p* < 0.05, *n* = 4, Tukey's multiple comparison test was used as *post-hoc* test).

### Average migratory speed is reduced after treatment with Hsp90 complex protein inhibitors and antibodies

**Figure 4:**
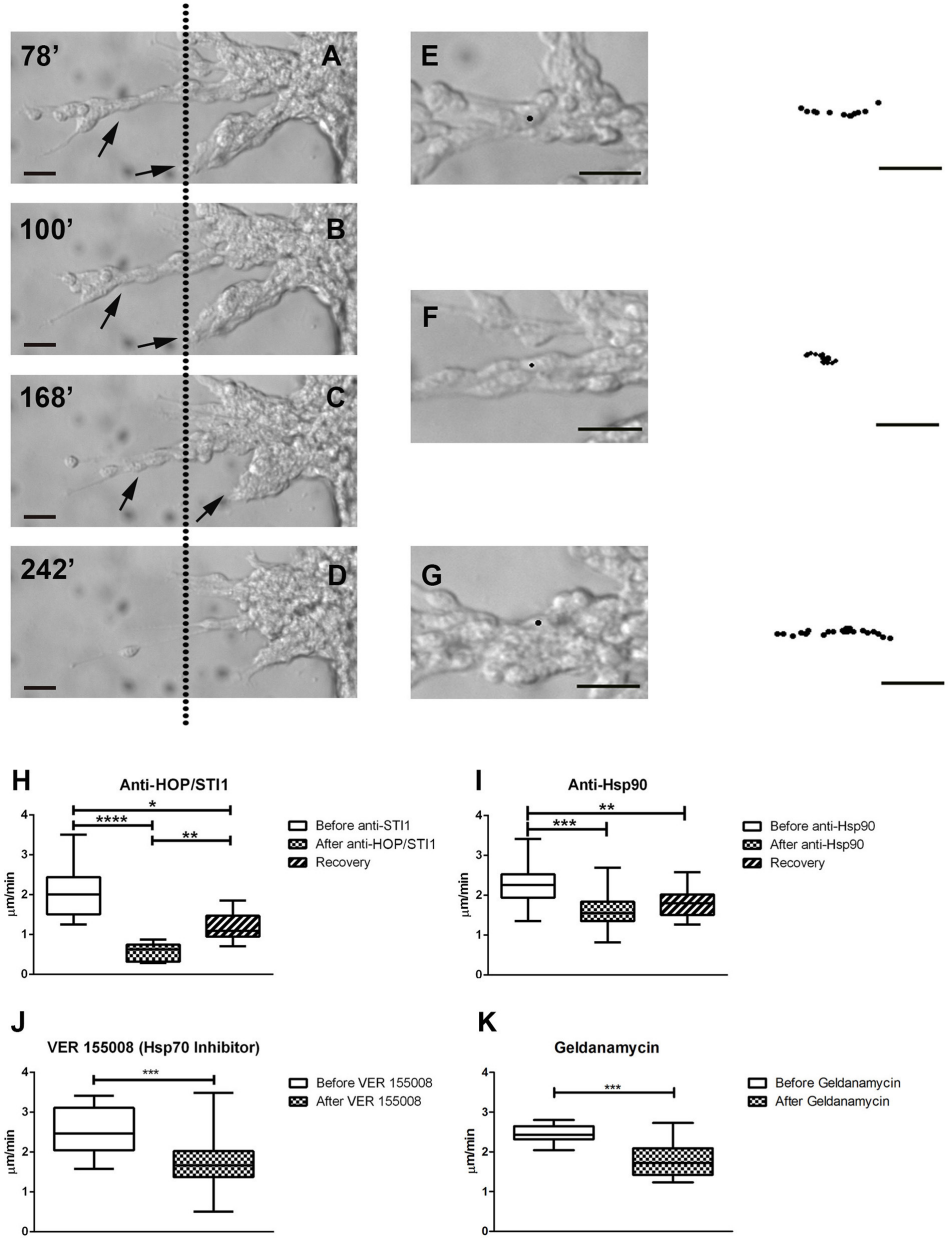
**Time-lapse videomicroscopy demonstrates collapse of migratory chains and decrease in average cell migratory speed**. In **A–D**, after anti-HOP/STI1 treatment, individual cells within chains are nearly indistinguishable, as the cells cluster into compact masses in the proximity of explants. Simultaneously, chains (arrows) start to collapse back to the explants. These responses were registered during the course of 164 min. Dotted line is used as a visual reference. The minutes indicate the time elapsed after the onset of the recording (**A**:78, **B**:100, **C**:168, and **D**:242 min). Individual cells were tracked before **(E)**, during **(F)** and after **(G)** anti-HOP/STI1 treatment, as can be seen in the bright-field images. The column in the far right depicts the cell trajectory during a given period of time (**E**, 24, **F**, 52, and **G**, 46 min) demonstrating that cell movement is inhibited in the presence of the antibody. Average cell speed is reduced in a significant manner after antibody treatment, as can be seen both with anti-HOP/STI1 (**H**, One way Anova, *p* < 0.0001) and anti-Hsp90 (**I**, One way Anova, *p* < 0.001), however this effect is only reversed after removal of anti-HOP/STI1 of the culture media (Anti-HOP/STI1, after anti-HOP/STI1 vs. recovery *p* < 0.01; Anti-Hsp90, after anti-Hsp90 vs. recovery, n.s.). A similar result was observed after exposure to VER155008 and Geldanamycin, Hsp70 and Hsp90 inhibitors, respectively (**J**, VER155008 unpaired *t*-test, *p* < 0.001; **K**, Geldanamycin unpaired *t*-test, *p* < 0.01) Scale bars: **(A–D)** 20 μm, **(E–G)** 20 μm. Tukey's multiple comparison test was used as *post-hoc* test after One way Anova analysis. ^*^p < 0.05, ^**^p < 0.01, ^***^p < 0.001, ^****^p < 0.0001.

We then performed time-lapse video microscopy experiments to observe in a detailed manner the effects of these antibodies and inhibitors on neuroblast migration. As previously described, in control conditions neuroblasts exhibit highly dynamic behavior, sliding over each other, which is a hallmark feature of chain migration. In these chains, cells were observed to move forward and backwards, thus the movement was bidirectional, however net growth was always away from the explant. When anti-HOP/STI1 or anti-Hsp90 was applied to the cultures, cell behavior was drastically modified. Overall movement was abolished and individual cells became nearly indistinguishable, due to the more compact organization within chains. Eventually, these chains tended to collapse back to the explants (Figures 4A–D, see also Supplementary video). After antibody treatment average migratory speed of cells was reduced in a significant manner. This effect was observed after both anti-HOP/STI1 (Figures 4E–H, One way ANOVA, Tukey's multiple comparison test used as *post-hoc, p* < 0.0001) and anti-Hsp90 exposure (Figure 4I, One way ANOVA, Tukey's multiple comparison test used as a *post-hoc, p* < 0.001), although the response after anti-HOP/STI1 exposure was even more drastic. Interestingly, only after removal of anti-HOP/STI1 from culture medium the average speed was partially recovered (Supplementary Video 1). A similar reduction in average speed was observed after treatment with inhibitors specific to Hsp90 (Figure 4K, Geldanamycin 100 nM, unpaired *t*-test, *p* < 0.01) and Hsp70 (Figure 4J, VER155008 10 nM, unpaired *t*-test, *p* < 0.001). Despite the decrease in the average speed, effects mediated by the inhibitors are not immediate as in the case of the antibodies, demanding more time to be apparent (Supplementary Videos 3, 4). Taken together, these results support the hypothesis that the inhibition of protein function, whether by antibodies or inhibitors, is responsible for the disruption of neuroblast migration.

### Immunofluorescence analysis of Hsp90 complex in non-permeabilized cultures

**Figure 5:**
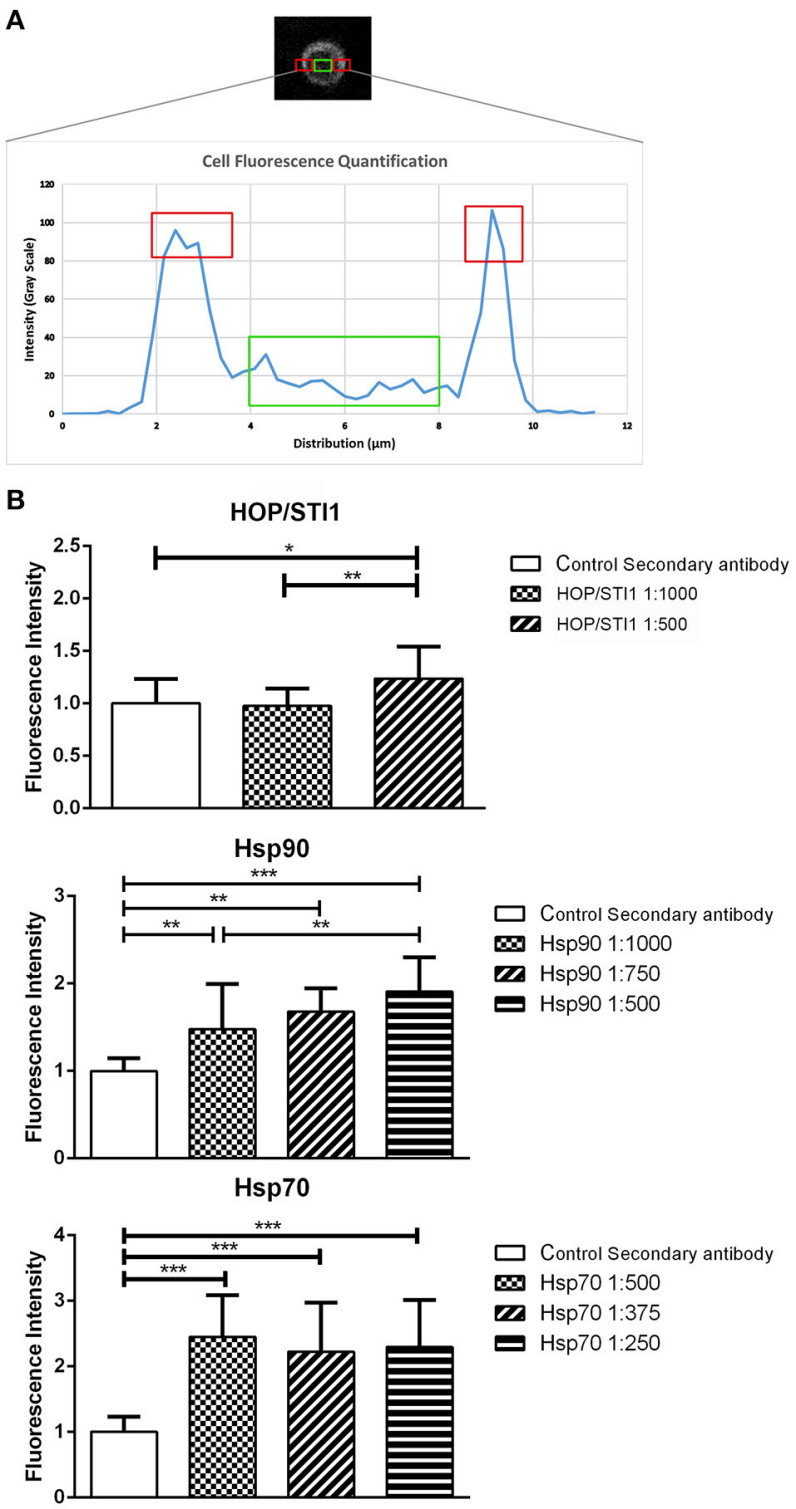
**Proteins from a chaperone heterocomplex are present in the cell membrane**. Live labeling of primary SVZ cultures detects antibody binding to cell membranes **(A)** plot that exemplifies how fluorescence analysis was performed in order to evaluate protein localization in single SVZ cells. Red areas correspond to the peak intensity signal detected from the cell membranes, while the green area depicts basal fluorescence level around the nucleus. **(B)** Quantification of fluorescence from primary SVZ cultures submitted to live labeling assays. Antibodies that bind to HOP/STI1, Hsp90 and Hsp70 generate fluorescence signal that is significant higher than all negative control groups and it is also dose dependent. One way Anova, Tukey multiple comparison test as *post-hoc*. ^*^p < 0.05, ^**^p < 0.01, ^***^p < 0.001.

We hypothesize that the function of the Hsp90 complex in mediating migration mechanisms is exerted extracellularly interfering with cell-substrate or cell-cell interactions. As seen before in Figure 2, prior permeabilization of neuroblast membranes with triton-X-100 affects the immunolabeling pattern of HOP/STI1, which becomes sparse. These findings suggest that HOP/STI1 could be present on cell membranes in addition to the extracellular pool already well characterized (Hajj et al., 2013). To investigate this, we analyzed HOP/STI1 immunofluorescence distribution in the membranes of SVZ primary cultures submitted to live labeling using the antibodies that bind to the proteins of the complex of chaperones. Single optical sections images obtained through confocal microscopy were analyzed in Image J software to assess fluorescence intensity. The plots generated reveal that while fluorescence in the intracellular region remains in a constant basal level, there is a peak signal from the sites corresponding to the cellular membranes (Figure 5A). Live labeling using antibodies against HOP/STI1, Hsp90, and Hsp70 resulted in an average cell membrane fluorescence that was significantly higher than control groups where only the secondary antibodies were used (Figure 5B). We observed that in the case of anti-HOP/STI1 and Hsp90, fluorescence intensity was also dose-dependent, an indicator that this effect is correlated to the specific binding of antibodies to the membranes. These results could be indicative that proteins of the complex are present in the cell surface.

### Chaperone/co-chaperone components of the Hsp90 complex were identified at the external membrane leaflet as well as in the conditioned medium of SVZ explants

**Figure 6:**
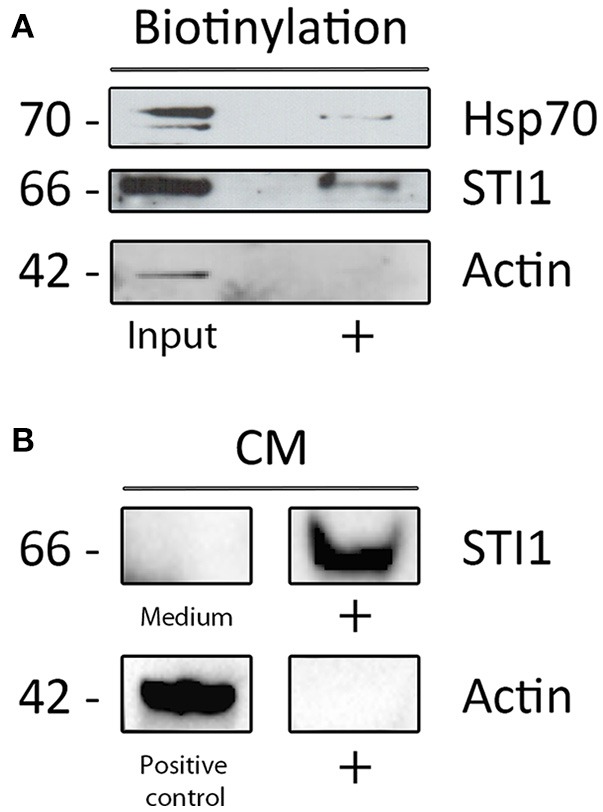
**Proteins from the heterocomplex are not only present in cell membranes, but also in conditioned medium. (A)** Both HOP/STI1 and Hsp70 are present in biotinylated cell membrane extracts from SVZ. As control, α-actin was not detected in the same fraction analyzed. **(B)** The co-chaperone HOP/STI1 is also present in conditioned medium (CM) collected from SVZ primary cultures, α-actin was not detected in the CM samples.

In order to confirm the presence of these proteins in cell surface or extracellular environment, dissociated SVZ cells were cultivated for 48–72 h and cell surface proteins subjected to chemical coupling to biotin. The incubation with biotin was performed at 4°C to ensure that the coupling reaction would happen only with the proteins outside the cell and no activated biotin could be internalized. Further, biotin reaction was directly performed over cells attached to the plastic culture dishes to avoid cell membrane disruption. Cellular extracts containing biotinylated proteins were affinity-purified with avidin-agarose matrices and further subjected to Western Blot analysis which revealed the cell surface presence of Hsp70 and HOP/STI1 (Figure 6A). Importantly, actin was not pulled-down by avidin-agarose beads, indicating absence of activated-biotin leaking to cytoplasm during coupling reaction. Further evidence of extracellular located HOP/STI1 was found in conditioned medium obtained from 48 h SVZ primary cultures. Western Blot analysis showed that HOP/STI1 was present in the concentrated conditioned medium (Figure 6B). Further, the absence of actin in the samples excludes the possibility that detected HOP/STI1 was originated from disrupted cells.

## Discussion

In this study, we hypothesized that proteins of the Hsp90 complex are involved with the regulation of neuroblast migration. We have described the expression of chaperone Hsp90 and co-chaperone HOP/STI1 in migratory neuroblasts from the subventricular zone. To evaluate their functional role in this biological context, we treated SVZ cells with antibodies and chemical inhibitors to Hsp90, Hsp70, and HOP/STI1 and observed that normal migratory behavior was disrupted. It is the first time that all these components of the Hsp90 complex were analyzed collectively in their role in neuronal migration. We also investigated a possible extracellular location of these proteins and showed their presence in the external leaflet of the plasmatic membranes and at least HOP/STI1 was detected in conditioned medium obtained from SVZ cultures. Therefore, we hypothesize that these proteins could be organized as a complex outside of the neuroblasts and perform an important role in the regulation of tangential neuroblast migration.

### Chaperones and co-chaperones affecting cell migration

Previous studies have shown that common mechanisms can regulate both neuronal and cancer cell migration, such as metalloproteinases (Lee et al., 2006; Akter et al., 2015), gangliosides (Miyakoshi et al., 2012; Ohkawa et al., 2015), tyrosine kinase, and integrin receptors (Anton et al., 2004; Sidera et al., 2008). A variety of chaperones and associated proteins have already been implicated in the regulation of cell migration (Sims et al., 2011; El Hamidieh et al., 2012). Our results suggest that Hsp90 and Hsp70, as well as co-chaperone HOP/STI1 are playing a functional role in neuroblast migration. One possibility is that these proteins might act as a complex, similar to the one described for the assembly and activity of the glucocorticoid receptor (Pratt et al., 2008), to influence migratory behavior. Although there is no definitive evidence to support this hypothesis, there is an indication that such a complex might be present in our model, since the individual inhibition of each protein yields similar results. Additionally, some of the studies associating chaperones and co-chaperones with cell migration also claim that this regulation occurs at the extracellular membrane surface (Sidera et al., 2008; Boroughs et al., 2011). The chaperones Hsp90 and Hsp70 appear to interact with different molecules both in the cell membrane and extracellular matrix, like tissue transglutaminase (tTG), the tyrosine kinase receptor HER-2 and matrix metalloproteinase-2 (MMP-2) and these interactions are important to allow cancer cell migration. In addition, HOP/STI1 has been involved with the migration and invasion of tumor cells (de Lacerda et al., 2016). Therefore, it has already been described that these proteins can exert non-cytoplasmic functions. Data obtained from biotinylation and live labeling experiments suggest that the elements of the Hsp90 complex are also localized to the cell membranes in our model. Additionally, we detected HOP/STI1 in conditioned medium samples, reinforcing the extracellular role of HOP/STI1 in this model. Some studies have described these proteins within the extracellular milieu employing conditioned medium obtained from cancer cells (Eustace and Jay, 2004; Erlich et al., 2007; Sims et al., 2011; de Lacerda et al., 2016), microglia (Fonseca et al., 2012) and astrocytes (Lima et al., 2007; Hajj et al., 2013). It has been demonstrated that chaperones Hsp90, Hsp70, and co-chaperone HOP/STI1 are released from the cell via exosomes (Lancaster and Febbraio, 2005; Mccready et al., 2010; Hajj et al., 2013). Another possibility is that this secretion could be mediated by a non-vesicular, lipid raft dependent mechanism (Broquet et al., 2003; Hunter-Lavin et al., 2004; Lancaster and Febbraio, 2005; Evdokimovskaya et al., 2010). Despite this evidence, it is not fully understood whether they are secreted as a complex or individual elements.

### Intracellular vs. extracellular role of chaperones and co-chaperones in the regulation of migration

The intracellular interaction of HOP/STI1 with the small GTPase Rnd1 found in PC12 cells interferes with actin polymerization and could be potentially important for neuroblast migration (De Souza et al., 2014). However, our time- lapse videomicroscopy data which analyzes the effect of antibody treatment on SVZ neuroblast migration shows that the decrease in migration velocity occurs within a time-frame of 30 min. These results suggest that the antibody function was exerted extracellularly, since when live-labeling with anti-HOP/STI1 was performed, no evidence of cytoplasmic labeling in SVZ cells was found within 1 h suggesting antibodies could not have an intracellular role.

Also, in a previous study (Miyakoshi et al., 2012) we observed that the immunoblockade of the 9-O-acetyl GD3 ganglioside, a glycolipid GPI anchored to the plasma membrane, caused effects to the neuroblast migration that are similar to the ones described in this study. We detected the same formation of cellular clusters, decrease of cell mobility, impaired distinction of individual cells within chains and retraction of these chains back to the explants. Thus, it seems plausible to argue that our current results are caused by inhibition of the antigens located in the cell surface.

### Possible mechanisms mediated by the extracellular chaperone complex regulating neuronal migration

It has been previously demonstrated that elements of this chaperone complex are able to regulate cell migration through the modulation of distinct mechanisms. The interaction of Hsp90 with the extracellular portion of receptors is suggested to be important for heterodimerization (Sidera et al., 2008) in cancer cells. Tyrosine kinases known to be client proteins to Hsp90 chaperones are amongst the possible targets for chaperone function in migration. In particular, ErbB4, which belongs to the HER-2 family of tyrosine kinase receptors has been implicated in neuroblast migration along the SVZ RMS and the corresponding knock-out models display migratory deficits (Anton et al., 2004).

Another extracellular role proposed consists in the activation of metalloproteinase-2 (Sims et al., 2011). Apparently, several chaperones and co-chaperones, including Hsp70 and HOP/STI1, are able to enhance binding of Hsp90 and consequently the activation of this metalloproteinase. The activity of this protease is directly related to the increased migration of breast cancer cells, probably by aiding extracellular matrix and basal membrane degradation.

## Conclusions

Our results showed that proteins of Hsp90 complex, such as HOP/STI1, Hsp90, and Hsp70 influence SVZ neuroblast migration. Additionally, similar results obtained with inhibitors or antibodies suggest that these different elements may act in a concerted fashion to regulate migration. Finally, the extracellular presence of some of the HSP90 components indicates that these proteins might perform their roles outside of the cell.

## Ethics statement

This study was carried out in accordance with the recommendations and guidelines determined by the National Council for Control of Animal Experimentation (CONCEA) in Brazil according to Law n° 11.794, issued in October 8, 2008 and decree n° 6.899, issued July 15, 2009. The protocol was approved by the Committee for Ethics In Use of Animals for Scientific Experimentation (CEUA) from the Center for Health Sciences (CCS) of the Federal University of Rio de Janeiro. This study was registered in CEUA under the number 033/15 and in CONCEA under 01200.001568/2013-87.

## Author contributions

LM and DC has contributed with design and execution of most experiments. LD has contributed with the biochemistry experiments. FL contributed with the experimental design and manuscript revision. VM contributed with reagents, design and manuscript revision. SZ contributed with reagents and helped direct biochemistry experiments. CH contributed with design, funding and writing this article together with LM and DC.

## Funding

CNPq, FAPERJ (E-26/010.002902/2014), FAPERJ-PRONEX, FAPESP. CAPES Master scholarship to DC and CNPq Doctorate scholarship to LM.

### Conflict of interest statement

The authors declare that the research was conducted in the absence of any commercial or financial relationships that could be construed as a potential conflict of interest.

## Supplementary material

In the “Before anti-STI1 treatment,” “Anti-STI1 treatment,” and “Reversion” videos, cultures were imaged during approximately 4 h and each frame is taken every 2 min. The “Before anti-Hsp90” and “Hsp90 treatment” videos were recorded during 2 h and 30 min, frames were captured every minute. In the “Before Geldanamycin” and “Geldanamycin 100 nm” videos, images were taken every 1.5 min during 3 h. In the “Before VER155008” and “VER155008 10 nM” videos, images were taken every minute during 3 h.
